# Burden and associated phenotypic characteristics of tuberculosis infection in adult Africans with diabetes: a systematic review

**DOI:** 10.1038/s41598-023-47285-4

**Published:** 2023-11-14

**Authors:** Davis Kibirige, Irene Andia-Biraro, Andrew Peter Kyazze, Ronald Olum, Felix Bongomin, Rose Mwanje Nakavuma, Phillip Ssekamatte, Reagan Emoru, Goretti Nalubega, Nyasatu Chamba, Kajiru Kilonzo, Sweetness Naftal Laizer, Lucy Elauteri Mrema, Willyhelmina Olomi, Lilian Tina Minja, Nyanda Elias Ntinginya, Issa Sabi, Philip C. Hill, Lindsey te Brake, Reinout van Crevel, Katrina Sharples, Julia Critchley

**Affiliations:** 1 Department of Medicine, Uganda Martyrs’ Hospital Lubaga, Kampala, Uganda; 2 Clinical Research Unit, Tuberculosis and Comorbidities Consortium, Kampala, Uganda; 3 Department of Medicine, Makerere University College of Health Sciences, Kampala, Uganda; 4 Department of Medicine, St. Francis Hospital Nsambya, Kampala, Uganda; 5 Department of Medical Microbiology and Immunology, Faculty of Medicine, Gulu University, Gulu, Uganda; 6 Department of Medicine, Kilimanjaro Christian Medical Centre, Moshi, Tanzania; 7 Department of Medicine, Kilimanjaro Christian Medical University College, Moshi, Tanzania; 8 National Institute for Medical Research-Mbeya Medical Research Centre, Mbeya, Tanzania; 9 Centre for International Health, Otago University, Dunedin, New Zealand; 10 Department of Pharmacy, Radboud Institute for Health Sciences, Radboud University Medical Centre, Nijmegen, The Netherlands; 11 Department of Internal Medicine and Radboud Centre for Infectious Diseases, Radboud University Medical Centre, Nijmegen, The Netherlands; 12 Centre for Tropical Medicine and Global Health, Nuffield Department of Medicine, University of Oxford, Oxford, UK; 13 Population Health Research Institute, St. George’s University of London, London, UK

**Keywords:** Immunology, Diseases, Endocrinology

## Abstract

Diabetes mellitus (DM) increases the risk of developing tuberculosis infection (TBI). However, the evidence on the burden and phenotypic characteristics of TBI in African patients with DM is limited. This study aimed to determine the prevalence and characterisation of TBI in native African patients living with DM. We searched PubMed, EMBASE, and African Journals Online for original studies reporting information on the prevalence and characteristics of TBI in adult Africans with DM. A forest plot was used to describe the pooled prevalence estimate of TBI and the corresponding 95% confidence intervals (CI). Six studies conducted in four African countries involving 721 participants with DM were included in this systematic review. The pooled prevalence estimate of TBI was 40% (95% CI 20–60%, I^2^ = 98.52%, p < 0.001). Age ≥ 40 years and glycated haemoglobin levels independently predicted TBI positivity in patients with DM in three studies. Africans with DM have a high prevalence of TBI, especially those who are older or with poorly controlled diabetes. This justifies the need for studies to explore how to screen and manage TBI to avert the progression to active TB disease.

## Introduction

Globally, Africa is experiencing a growing dual burden of diabetes mellitus (DM) and tuberculosis (TB)^1,2^. The International Diabetes Federation estimates that the greatest future increase in the prevalence of DM over the next few decades will occur in Africa^1^. Most African countries also have a high TB burden^2^. This high dual DM and TB burden in most African countries poses significant public health challenges.

Tuberculosis infection (TBI) is a subclinical infection of mycobacterial tuberculosis (MTB) infection which is defined based on an individual’s cellular immune response to the MTB bacilli^3^. Approximately, 5–15% of individuals infected with MTB progress to active TB disease during the first 2–5 years^4,5^. Therefore, optimal early screening and management of TBI in high-risk individuals, such as people living with HIV, adult and paediatric contacts of patients with active TB disease, and patients with DM are justified to avert the risk of progression to TB disease^6^.

People with DM are at increased risk of developing both TBI and active TB disease due to impaired humoral and cellular immunity^7^. Two recent systematic reviews and meta-analyses of TBI in patients with DM reported an increased risk of TBI in patients with DM^8,9^. However, no study conducted on African patients with DM and TBI was included in either study. This is probably because both systematic reviews and meta-analyses included only studies with an additional control group of participants without DM.

Despite considerable information available on the burden of active TB disease in African patients with DM^10–13^, the evidence on the prevalence of TBI and its related phenotypic characteristics in Africans with DM is limited. To address this gap, we conducted this systematic review to determine the prevalence of TBI in native African patients living with DM in addition to the related sociodemographic, anthropometric, and metabolic characteristics.

## Methods

This systematic review was conducted according to the criteria outlined in the Preferred Reporting Items for Systematic Reviews and Meta-Analyses (PRISMA) statement^14^. The PRISMA checklist is available as an online Supplemental Table 1. The study protocol was registered in the PROSPERO International Prospective Register of systematic reviews (CRD42023414927).

**Table 1:** Characteristics and the findings of all eligible original studies.

Last name of 1st author, year of publication, and country	Study design and setting	Number of participants	Participant characteristics (for patients with diabetes)	Diagnostic criteria of TBI	Prevalence of TBI
A: East African studies (n = 3)					
Kazibwe A et al., 2023, Uganda	Tertiary healthcare facility Cross-sectional	185 participants with a known history of diabetes	Median (IQR) HbA1c, %: 8.4 (7.1–9.9) Median (IQR) BMI, kg/m^2^: 28.6 (24.8–32.9) Proportion of alcohol ingestion: 17.8% Proportion of smokers: 3.8% Median (IQR) duration of diabetes, months: 9 (5–20) Proportion of females: 78.4% Median (IQR) age, years: 50 (42–59)	QFT-GIT	57.4%
Andia-Biraro I et al., 2015, Uganda	Tertiary healthcare facility Case–control	103 participants with newly diagnosed diabetes (cases) and 201 age- and gender-matched healthy household contacts (controls)	Median (IQR) HbA1c, %: 9.9 (8.4–11.1) Median (IQR) FBG, mmol/L: 13.4 (10.2–22.3) Mean (SD) BMI, kg/m^2^: 27.9 (9.5) Proportion of females: 69%	QFT-GIT and chest X-ray	73% for cases and 71% for controls
Smith AGC et al., 2022, Ethiopia	Tertiary healthcare facilities Cross-sectional	31 (patients with newly diagnosed diabetes and household contacts with TB cases)	Median BMI, kg/m^2^: 26.1 (23.7–30.4) Prevalence of HIV comorbidity: 9.7% Proportion of alcohol ingestion: 9.7% Proportion of smokers: 9.7% Proportion of females: 64.5% Median (IQR) age: 44.6 (40.3–50.4)	QFT-GIT	87.1%
B: West African studies (n = 2)					
Chukwudi UK et al., 2020, Nigeria	Tertiary healthcare facilities Case–control	240 participants with a known history of diabetes as cases and 240 healthy controls without diabetes and HIV	–	TST and IGRA (T-SPOT TB)	25% with TST and 13.8% with IGRA for the cases and 2.5% and 4% with IGRA and TST, respectively for the controls
Adewole OO et al., 2015, Nigeria	Tertiary healthcare facility Case–control	60 participants with a known history of diabetes and 55 age-, sex-, and social class-matched healthy controls	–	QFT GIT	37% for cases and 20% for the controls
C: North African studies (n = 1)					
Agha MA et al., 2020, Egypt	Tertiary healthcare facility Cross-sectional	102 participants with a known history of diabetes	Mean (SD) HbA1c, %: 8.1 (1.7) Mean (SD) BMI, kg/m^2^: 35.1 (5.5) Proportion of smokers: 31.4% Mean (SD) duration of diabetes, years: 7.5 (6.0) Proportion of females: 45.1% Mean (SD) age, years: 50 (10.1)	QFT GIT and TST	8.8% with TST and 21.6% with QFT GIT

### Search strategy

We searched EMBASE, PubMed, and the African Journals Online databases for any published studies and conference proceedings from 1980 to April 2023. The database search was performed in April 2023. The following search terms were used: ‘diabetes mellitus’/exp OR ‘diabetes mellitus’ OR ‘diabetes’/exp OR ‘diabetes’ AND ‘latent tuberculosis’ OR ‘tuberculosis infection’ OR ‘ltbi’ OR ‘ltb’ OR ‘TBI’ AND ‘angola’ OR ‘benin’ OR ‘botswana’ OR ‘burkina faso’ OR ‘burundi’ OR ‘cameroon’ OR ‘cape verde’ OR ‘central african republic’ OR ‘chad’ OR ‘comoros’ OR ‘democratic republic congo’ OR ‘djibouti’ OR ‘egypt’ OR ‘equatorial guinea’ OR ‘eritrea’ OR ‘eswatini’ OR ‘lesotho’ OR ‘ethiopia’ OR ‘gabon’ OR ‘gambia’ OR ‘ghana’ OR ‘guinea-bissau’ OR ‘cote d ivoire’ OR ‘kenya’ OR ‘liberia’ OR ‘libyan arab jamahiriya’ OR ‘madagascar’ OR ‘malawi’ OR ‘mali’ OR ‘mauritania’ OR ‘mauritius’ OR ‘mayotte’ OR ‘morocco’ OR ‘mozambique’ OR ‘namibia’ OR ‘niger’ OR ‘nigeria’ OR ‘congo’ OR ‘reunion’ OR ‘rwanda’ OR ‘saint helena’ OR ‘sao tome and principe’ OR ‘senegal’ OR ‘seychelles’ OR ‘sierra leone’ OR ‘somalia’ OR ‘south africa’ OR ‘south sudan’ OR ‘sudan’ OR ‘tanzania’ OR ‘togo’ OR ‘tunisia’ OR ‘uganda’ OR ‘western sahara’ OR ‘zambia’ OR ‘zimbabwe’ OR ‘africa’.

In addition, a Google Scholar search and review of the references of all included articles was performed to identify any additional original articles and conference proceedings. We restricted the search and selection to only articles published in English.

### Study selection criteria

The preliminary screening of titles and abstracts to identify eligible articles was done independently by three reviewers (DK, IAB, and RO). This was followed by removing all duplicates. After the initial screening, full texts of the potentially eligible studies were retrieved and closely reviewed for eligibility by two reviewers (DK and RO).

The inclusion criteria of studies were: Cross-sectional, case–control, cohort studies, and randomised clinical trials published in English at any time reporting information on the prevalence and associated characteristics of TBI in native adult African patients with DM. In cases of disagreements, a fourth independent reviewer (APK) was used to resolve them. We excluded review articles, case series, and research articles published in languages other than English.

### Data extraction

After identifying the eligible original studies, relevant study information was extracted and recorded in a Microsoft Excel 2016 form. The information of interest that was extracted included the second name of the first author, the year the study was published, the country and region of Africa where the study was performed (Eastern, Western, Central, Southern, and Northern), number of study participants, study design and setting, mean (standard deviation or SD) or median (inter-quartile range or IQR) age and duration of diabetes, the proportion of females, smoking status, diabetes therapy used (oral agents and insulin), the proportion of participants with HIV comorbidity, socioeconomic status, area of residence, education status, mean (SD) or median (IQR) body mass index (BMI), glycated haemoglobin (HbA1c), and fasting blood glucose (FBG) of the participants, the screening test(s) used to diagnose TBI, the prevalence of TBI based on a tuberculin skin test (TST) and/or Interferon Gamma Release Assay (IGRA) as the screening tests.

### Operational definitions

All included studies defined TBI based on a positive TST and/or IGRA test. Patients with DM were either those with a known history of DM and receiving glucose-lowering therapy from outpatient diabetes or endocrinology clinic or newly diagnosed following internationally recognised guidelines for diagnosing DM.

### Assessment of quality of studies

The quality of all eligible studies included in the systematic review were assessed using the Hoy tool^15^. This was done independently by one author (APK).

### Study outcome

The study outcome was the prevalence of TBI as determined by either of the screening tests (TST and/or IGRA) and the sociodemographic, clinical, anthropometric, and metabolic characteristics of African patients with confirmed TBI.

### Data analysis

All analyses were performed using STATA V.16.0 statistical software (Stata Corp, USA). The prevalence estimates of TBI were described using a forest plot. Heterogeneity was assessed using the I^2^ value. A pooled estimate of prevalence was determined using a random-effect model meta-analysis with the DerSimonian and Laird method pooling random-effect estimates^16^. The planned meta-regression was not carried out due to the small number of studies. A narrative review was used to describe the phenotypic characteristics of the patients with TBI.

## Results

**Figure 1:**
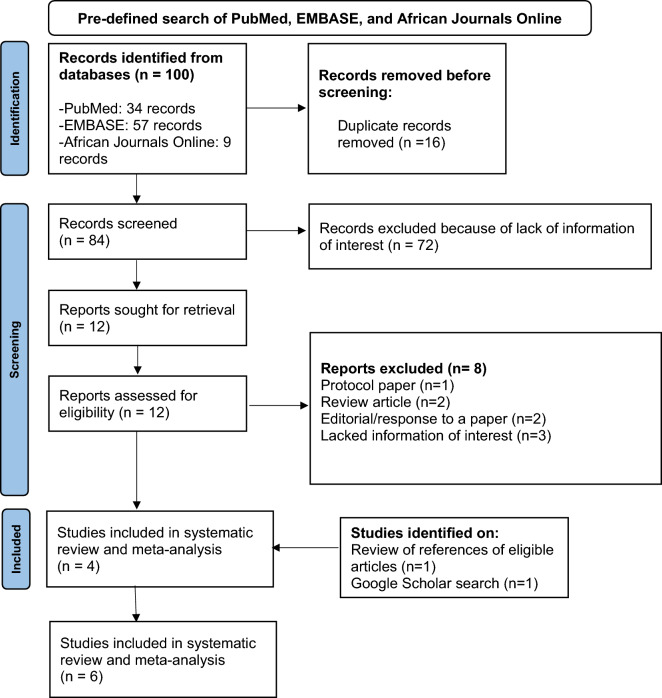
PRISMA flow diagram of selection of eligible studies.

The literature search returned a total of 100 articles (Fig. 1). From these, 16 duplicates were removed. Titles and abstracts of the remaining 84 articles were reviewed, and 12 were identified for full-text retrieval. Of these, eight were excluded, and the remaining four articles were included in the systematic review. The eight excluded articles included one protocol paper, two review articles, two editorials, and three articles that lacked information on the prevalence of TBI and associated characteristics. On hand-searching the references of the four articles, one published conference proceeding containing the relevant information was identified and also included. One published article was also identified by searching Google Scholar, making a total of six eligible articles included in the systematic review.

### Characteristics of included studies

Table 1 summarises the characteristics of all studies included in the systematic review.

The included studies were cross-sectional (n = 3, 50%)^17–19^ and case–control (n = 3, 50%) design^20–22^. Half of the studies were performed in Eastern African countries (n = 3, 50%)^18,19,21^ with two (33.3%) conducted in Western Africa (Nigeria)^20,22^, and one (16.7%) conducted in Northern Africa (Egypt)^17^.

All the studies defined the presence of TBI based on a positive IGRA test (QuantiFERON^®^ Gold In Tube or QFT-GIT and T-SPOT TB)^17–22^. Only two studies diagnosed TBI based on a positive TST and IGRA test^17,22^.

### Characteristics of study participants

Table 1 summarises the characteristics of all study participants.

The six studies had a total of 721 participants ranging from 31 participants in Ethiopia^19^ to 240 participants in Nigeria^22^. The proportion of females ranged from 45.1% in Egypt^17^ to 78.4% in Uganda^18^. Two studies recruited participants with newly diagnosed diabetes^19,21^ while the rest recruited participants with a known history of DM^17,18,20,22^.

Information on the mean (SD) or median (IQR) age and BMI of the study participants was reported by four studies^17–19,21^, with the median (IQR) age ranging from 44.6 (40.3–50.4) years in Ethiopia^19^ to 50 (42–59) years in Uganda^18^ and the BMI ranging from 26.1 (23.7–30.4) kg/m^2^ in Ethiopia^19^ to 35.1 ± 5.5 kg/m^2^ in Egypt^17^. Information on the status of long-term glycaemic control was reported in three studies conducted in Egypt and Uganda with the mean (SD) and median (IQR) HbA1c being 8.1 (1.7) %, 8.4 (7.1–9.9) %, and 9.9 (8.4–11.1) %, respectively^17,18,21^. Only one study provided information on the prevalence of HIV infection in patients with DM, reporting a prevalence of 9.7%^19^.

### Assessment of study quality

The assessment of the quality of studies is summarised in Supplementary Table 2.

Of the six studies included in the systematic review, four (66.7%) had a low risk of bias^17–19,22^. Because two studies were published conference proceedings without available full texts, we could not effectively assess their quality^20,21^.

### Prevalence of TBI

**Figure 2:**
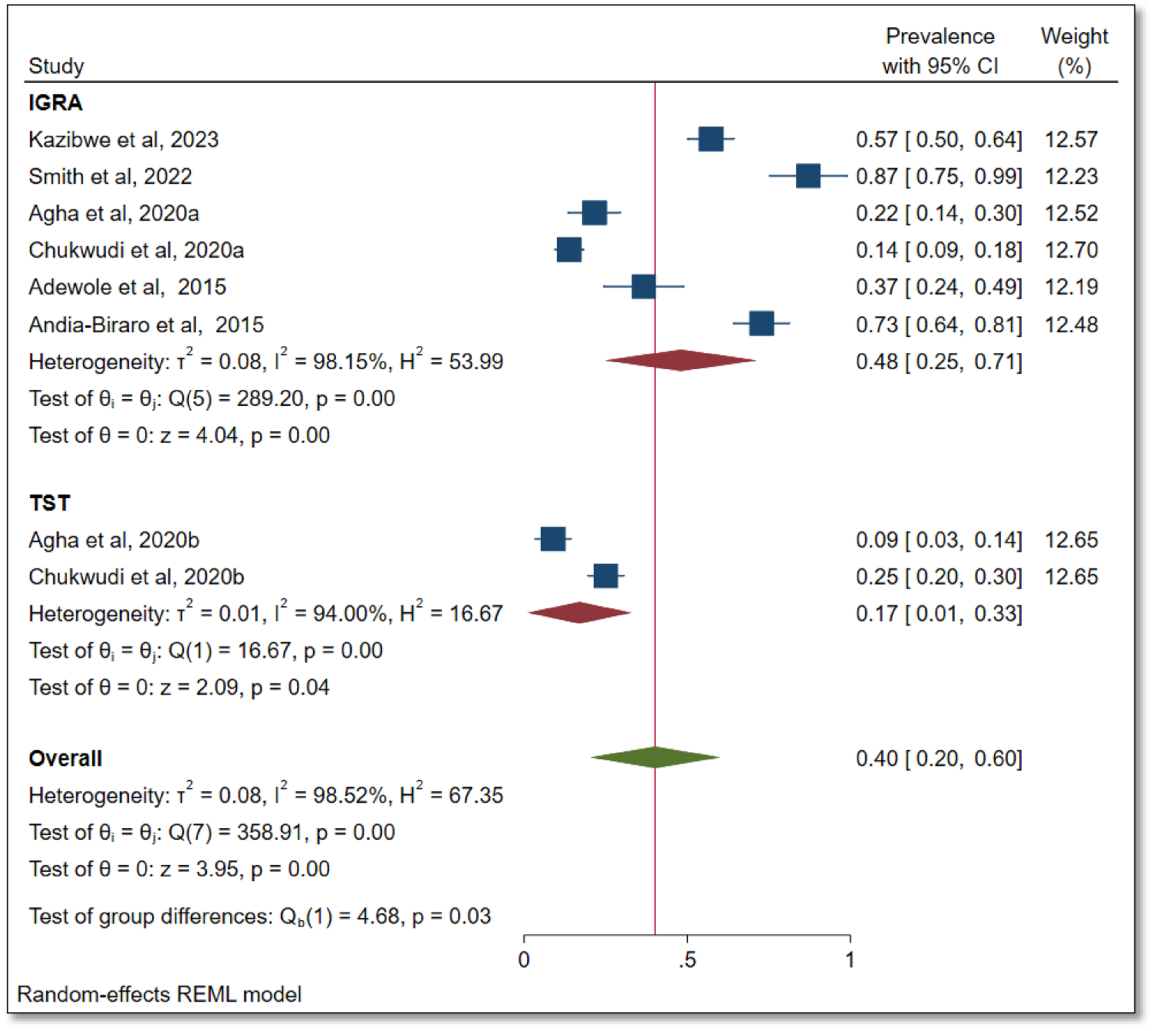
Prevalence of latent TB infection based on the screening test used.

The prevalence of TBI based on the screening test used is summarised as a forest plot in Fig. 2. Based on the IGRA or QFT GIT (n = 6 studies, 100%) and TST alone (n = 2 studies, 33.3%), the pooled prevalence estimates of TBI were 48% (95% CI 25–71%, I^2^ = 98.15%, P < 0.001) and 17% (95% 10–33%, I^2^ = 94.00%, p < 0.001), respectively. The overall pooled prevalence of TBI in this study population was 40% (95% CI 20–60%, I^2^ = 98.52%, p < 0.001).

### Phenotypic characteristics of patients with diabetes and TBI

Information on the sociodemographic, clinical, anthropometric, and metabolic characteristics of participants with and without DM and TBI comorbidity was reported by four studies^17–20^. Compared to those without DM and TBI comorbidity, participants with DM and TBI were more likely to have a higher median age, longer duration of DM, higher fasting blood glucose (FBG), and glycated haemoglobin (HbA1c) levels^17,19,20^.

Three of the four studies carried out multivariable logistic regression to identify predictors of TBI positivity and all three studies reported an age ≥ 40 years and HbA1c levels to be independently associated with TBI positivity in patients with DM^17,19,20^. In one cross-sectional study conducted on 185 adult Ugandan patients with long-standing DM and receiving outpatient diabetes care from a tertiary hospital, treatment with calcium channel blockers and pregabalin were independent predictors of TBI^18^.

None of the above studies reported evidence of an association between sex, BMI, HIV co-infection, the use of metformin therapy, and TBI.

## Discussion

To our knowledge, this is the first systematic review to document the burden and associated characteristics of TBI in adult native African patients with DM. We report a high burden of TBI in four adult African populations with DM. Participants with DM aged ≥ 40 years and with suboptimal glycaemic control (HbA1c > 7%) had increased odds of having TBI in three studies.

A comparable high prevalence of TBI in adult patients with DM has also been reported in some studies conducted in Asia^23–25^. As part of the multi-site Tuberculosis and Diabetes Mellitus (TANDEM) study, the prevalence of TBI in 651 Indonesians with DM screened using QFT GIT was 38.9%^24^. In another study that used TST as the screening test in 404 Malaysian patients with DM, the prevalence of TBI was 28.5%. Smoking was the only independent risk factor of TBI in this study, regardless of the glycaemic profile^25^.

In the Prognostic Evaluation of Diagnostic IGRAs Consortium (PREDICT) multi-site study that screened 9157 recent immigrants to the UK from high TB burden countries and contacts of active TB cases, the prevalence of TBI based on a positive QFT GIT test in 238 patients with DM was 31.5%^26^. About 12% of the screened study population were of black African ancestry and had the highest IGRA positivity rate (37%) compared with other non-Caucasian ethnicities^26^.

A high prevalence of TBI of 51.3% was reported in 600 Mexicans with type 2 diabetes and no prior history of TB. Contrary to what is used in most studies as the diagnostic cut-off point for TBI which is a TST of ≥ 10 mm, this study used a TST value of ≥ 5 mm to define the presence of TBI. This could explain the high prevalence of TBI reported by this study^27^.

Regarding predictors of TBI in patients with DM, similar to what was reported in the studies conducted in Egyptian^17^, Ethiopian^19^, and Nigerian^20^ patients with DM and TBI, being older (≥ 50 years) was associated with increased odds of having TBI in 1120 Taiwanese patients with DM. The prevalence of TBI in this study based on a positive QFT GIT was 21.5%^23^. This increased risk of TBI noted with increasing age may be due to the age-related reduction in the cellular and humoral immunity^28^.

Similar to what was reported in the study by Agha et al.^17^ and Adewole et al.^20^ in Egyptian and Nigerian participants with DM and TBI, HbA1c > 7% in addition to living with a relative with TB, having been in prison, and having hemoglobin values > 14 g/dL independently predicted TBI in the study conducted in Mexican patients with DM^27^. A study by Koesoemadinata et al. also reported living in crowded households as a key predictor of TBI in Indonesian patients with DM^24^.

While there is evidence on how to manage TBI in HIV-infected patients, evidence of its management in patients with DM is still lacking. This justifies the need for interventional studies to provide compelling evidence on how to optimally screen and manage TBI in this high-risk patient population and, therefore, avert the risk of progression to active TB disease.

To address the clinical gap regarding the lack of robust clinical evidence of which therapeutic regimen should be used to manage TBI in patients with DM in addition to assessing its safety, the Preventive Treatment Of Latent Tuberculosis Infection In People With Diabetes Mellitus (PROTID) study (clinicaltrials.gov NCT04600167) being conducted currently in Uganda and Tanzania has been randomising adult patients with confirmed TBI (by TST and/or IGRA) and DM to either 3 months of isoniazid-rifapentine (3HP) or placebo. The participants are being followed up for 2 years to determine the incidence of probable or definite TB disease^29^. The study is also enrolling a parallel cohort of adult patients without TBI and following them up for 2 years to determine the incidence of definite or probable active TB disease. The PROTID study will provide valuable information about the burden and determinants of TBI in a large cohort of East African patients with DM and will also investigate the diagnostic concordance of the TST and IGRA as screening tests for TBI in these two East African populations with DM with a broad aim of informing clinical practice and policy in Africa.

### Strengths and limitations of the systematic review

This is the first systematic review to investigate the burden and associated characteristics of TBI in African patients with DM. Most of the included studies had a low risk of bias.

Despite this strength, it has some limitations. Most of the studies recruited a small number of participants and provided limited information on the phenotypic characteristics of the recruited participants. Two of the included studies were conference proceedings without available full texts to provide detailed information on the sociodemographic, anthropometric, and metabolic characteristics of the study participants. All studies were conducted in tertiary healthcare facilities and this may result in a biased estimate of the prevalence of TBI in people with DM. The studies included in this systematic review were of considerable heterogeneity and, therefore, the overall effect size should be interpreted with caution.

## Conclusion

This systematic review reported a high prevalence of TBI in African patients with DM especially those aged ≥ 40 years and with poor glycaemic control. This finding supports the approach of targeted screening and management of TBI in this high-risk population. There is a need for interventional trials to inform clinicians about the most appropriate and cost-effective approach to screening and managing TBI in adult Africans with DM as a feasible TB control strategy.

### Supplementary Information

Supplementary Information.

Supplementary Tables.

## Data Availability

The data file used in this systematic review is attached as a supplementary file. All studies included in this systematic review are published and freely accessible online.
