# Supplementary material for: Burden and associated phenotypic characteristics of tuberculosis infection in adult Africans with diabetes: a systematic review
Source: Sci Rep. 2023 Nov 14;13:19894. doi: 10.1038/s41598-023-47285-4 (PMC10645877; doi:10.1038/s41598-023-47285-4)
Supplement: Supplementary file 2 — Supplementary Tables. [file 41598_2023_47285_MOESM2_ESM.pdf]

Supplementary table 1. PRISMA checklist for the systematic review and meta-analysis

Section and Topic	Item #	Checklist item	Page where item is reported
**TITLE**			
Title	1	Identify the report as a systematic review.	1
**ABSTRACT**			
Abstract	2	See the PRISMA 2020 for Abstracts checklist.	3
**INTRODUCTION**			
Rationale	3	Describe the rationale for the review in the context of existing knowledge.	4-5
Objectives	4	Provide an explicit statement of the objective(s) or question(s) the review addresses.	5
**METHODS**			
Eligibility criteria	5	Specify the inclusion and exclusion criteria for the review and how studies were grouped for the syntheses.	6
Information sources	6	Specify all databases, registers, websites, organisations, reference lists and other sources searched or consulted to identify studies. Specify the date when each source was last searched or consulted.	5-6
Search strategy	7	Present the full search strategies for all databases, registers and websites, including any filters and limits used.	5-6
Selection process	8	Specify the methods used to decide whether a study met the inclusion criteria of the review, including how many reviewers screened each record and each report retrieved, whether they worked independently, and if applicable, details of automation tools used in the process.	6
Data collection process	9	Specify the methods used to collect data from reports, including how many reviewers collected data from each report, whether they worked independently, any processes for obtaining or confirming data from study investigators, and if applicable, details of automation tools used in the process.	6-7
Data items	10a	List and define all outcomes for which data were sought. Specify whether all results that were compatible with each outcome domain in each study were sought (e.g. for all measures, time points, analyses), and if not, the methods used to decide which results to collect.	7
	10b	List and define all other variables for which data were sought (e.g. participant and intervention characteristics, funding sources). Describe any assumptions made about any missing or unclear information.	7
Study risk of bias assessment	11	Specify the methods used to assess risk of bias in the included studies, including details of the tool(s) used, how many reviewers assessed each study and whether they worked independently, and if applicable, details of automation tools used in the process.	7
Effect measures	12	Specify for each outcome the effect measure(s) (e.g. risk ratio, mean difference) used in the synthesis or presentation of results.	7
Synthesis methods	13a	Describe the processes used to decide which studies were eligible for each synthesis (e.g. tabulating the study intervention characteristics and comparing against the planned groups for each synthesis (item #5)).	7-8
	13b	Describe any methods required to prepare the data for presentation or synthesis, such as handling of missing summary statistics, or data conversions.	7-8
	13c	Describe any methods used to tabulate or visually display results of individual studies and syntheses.	7-8
	13d	Describe any methods used to synthesize results and provide a rationale for the choice(s). If meta-analysis was performed, describe the model(s), method(s) to identify the presence and extent of statistical heterogeneity,	7-8

Section and Topic	Item #	Checklist item	Page where item is reported
		and software package(s) used.	
	13e	Describe any methods used to explore possible causes of heterogeneity among study results (e.g. subgroup analysis, meta-regression).	8
	13f	Describe any sensitivity analyses conducted to assess robustness of the synthesized results.	Not done
Reporting bias assessment	14	Describe any methods used to assess risk of bias due to missing results in a synthesis (arising from reporting biases).	8
Certainty assessment	15	Describe any methods used to assess certainty (or confidence) in the body of evidence for an outcome.	8
**RESULTS**			
Study selection	16a	Describe the results of the search and selection process, from the number of records identified in the search to the number of studies included in the review, ideally using a flow diagram.	8
	16b	Cite studies that might appear to meet the inclusion criteria, but which were excluded, and explain why they were excluded.	8
Study characteristics	17	Cite each included study and present its characteristics.	8-9
Risk of bias in studies	18	Present assessments of risk of bias for each included study.	9
Results of individual studies	19	For all outcomes, present, for each study: (a) summary statistics for each group (where appropriate) and (b) an effect estimate and its precision (e.g. confidence/credible interval), ideally using structured tables or plots.	10-11
Results of syntheses	20a	For each synthesis, briefly summarise the characteristics and risk of bias among contributing studies.	9
	20b	Present results of all statistical syntheses conducted. If meta-analysis was done, present for each the summary estimate and its precision (e.g. confidence/credible interval) and measures of statistical heterogeneity. If comparing groups, describe the direction of the effect.	9-10
	20c	Present results of all investigations of possible causes of heterogeneity among study results.	Not done
	20d	Present results of all sensitivity analyses conducted to assess the robustness of the synthesized results.	Not done
Reporting biases	21	Present assessments of risk of bias due to missing results (arising from reporting biases) for each synthesis assessed.	NA
Certainty of evidence	22	Present assessments of certainty (or confidence) in the body of evidence for each outcome assessed.	10
**DISCUSSION**			
Discussion	23a	Provide a general interpretation of the results in the context of other evidence.	11-13
	23b	Discuss any limitations of the evidence included in the review.	13
	23c	Discuss any limitations of the review processes used.	13
	23d	Discuss implications of the results for practice, policy, and future research.	14
**OTHER INFORMATION**			
Registration and protocol	24a	Provide registration information for the review, including register name and registration number, or state that the review was not registered.	5
	24b	Indicate where the review protocol can be accessed, or state that a protocol was not prepared.	Protocol was not prepared
	24c	Describe and explain any amendments to information provided at registration or in the protocol.	None
Support	25	Describe sources of financial or non-financial support for the review, and the role of the funders or sponsors in the review.	15

Section and Topic	Item #	Checklist item	Page where item is reported
Competing interests	26	Declare any competing interests of review authors.	14
Availability of data, code and other materials	27	Report which of the following are publicly available and where they can be found: template data collection forms; data extracted from included studies; data used for all analyses; analytic code; any other materials used in the review.	14

Supplementary Table 2. Risk of bias Assessment of the studies

Risk of bias item and Criteria for answers	Agha et al 2020	Kazibwe et al 2023	Smith et al 2022	Adewole et al. 2015	Andia-Biraro I et al. 2019	Chukwudi et al. 2020
**A: EXTERNAL VALIDITY**						
1. Was the study's target population a close representation of the national population in relation to relevant variables, e.g. age, sex, occupation?	Yes (LOW RISK): The study's target population was a close representation of the national population*	Yes (LOW RISK): The study's target population was a close representation of the national population*	Yes (LOW RISK): The study's target population was a close representation of the national population*	Yes (LOW RISK): The study's target population was a close representation of the national population*	Yes (LOW RISK): The study's target population was a close representation of the national population*	Yes (LOW RISK): The study's target population was a close representation of the national population*
2. Was the sampling frame a true or close representation of the target population?	Yes (LOW RISK): The sampling frame was a true or close representation of the target population	Yes (LOW RISK): The sampling frame was a true or close representation of the target population	Yes (LOW RISK): The sampling frame was a true or close representation of the target population	Yes (LOW RISK): The sampling frame was a true or close representation of the target population	Yes (LOW RISK): The sampling frame was a true or close representation of the target population	Yes (LOW RISK): The sampling frame was a true or close representation of the target population
3. Was some form of random selection used to select the sample, OR, was a census undertaken?	Yes (LOW RISK): Some form of random selection was used to select the sample	Yes (LOW RISK): Some form of random selection was used to select the sample	Yes (LOW RISK): Some form of random selection was used to select the sample	Cannot assess that because only an abstract was available	Cannot assess that because only an abstract was available	Yes (LOW RISK): Some form of random selection was used to select the sample
4. Was the likelihood of non-response bias minimal?	Yes (LOW RISK): The response rate for the study was adequate	Yes (LOW RISK): The response rate for the study was adequate	Yes (LOW RISK): The response rate for the study was adequate	Cannot assess that because only an abstract was available	Cannot assess that because only an abstract was available	Yes (LOW RISK): The response rate for the study was adequate
**B: INTERNAL VALIDITY**						
5. Were data collected directly from the subjects (as opposed to a proxy)?	Yes (LOW RISK): All data were collected directly from the subjects.	Yes (LOW RISK): All data were collected directly from the subjects.	Yes (LOW RISK): All data were collected directly from the subjects.	Cannot assess that because only an abstract was available	Cannot assess that because only an abstract was available	Yes (LOW RISK): All data were collected directly from the subjects.
6. Was an acceptable case definition used in the study?	Yes (LOW RISK): An acceptable case definition was used.	Yes (LOW RISK): An acceptable case definition was used.	Yes (LOW RISK): An acceptable case definition was used.	Cannot assess that because only an abstract was available	Cannot assess that because only an abstract was available	Yes (LOW RISK): An acceptable case definition was used.
7. Was the study instrument that measured the parameter of interest shown to have reliability and validity (if necessary)?	Yes (LOW RISK): The study instrument has been shown to have reliability and validity	Yes (LOW RISK): The study instrument has been shown to have reliability and validity	Yes (LOW RISK): The study instrument has been shown to have reliability and validity	Cannot assess that because only an abstract was available	Cannot assess that because only an abstract was available	Yes (LOW RISK): The study instrument has been shown to have reliability and validity
8. Was the same mode of data collection used for all subjects?	Yes (LOW RISK): The same mode of data collection was used for all subjects.	Yes (LOW RISK): The same mode of data collection was used for all subjects.	Yes (LOW RISK): The same mode of data collection was used for all subjects.	Cannot assess that because only an abstract was available	Cannot assess that because only an abstract was available	Yes (LOW RISK): The same mode of data collection was used for all subjects.

9. Was the <u>length of the shortest prevalence period</u> for the parameter of interest appropriate?	Yes (LOW RISK): The shortest prevalence period for the parameter of interest was appropriate	Yes (LOW RISK): The shortest prevalence period for the parameter of interest was appropriate	Yes (LOW RISK): The shortest prevalence period for the parameter of interest was appropriate	Cannot assess that because only an abstract was available	Cannot assess that because only an abstract was available	Yes (LOW RISK): The shortest prevalence period for the parameter of interest was appropriate
10.Were the <u>numerator(s) and denominator(s)</u> for the parameter of interest appropriate?	Yes (LOW RISK): The paper presented appropriate numerator(s) AND denominator(s) for the parameter of interest	Yes (LOW RISK): The paper presented appropriate numerator(s) AND denominator(s) for the parameter of interest	Yes (LOW RISK): The paper presented appropriate numerator(s) AND denominator(s) for the parameter of interest	Cannot assess that because only an abstract was available	Cannot assess that because only an abstract was available	Yes (LOW RISK): The paper presented appropriate numerator(s) AND denominator(s) for the parameter of interest
**C: SUMMARY ITEM ON THE OVERALL RISK OF STUDY BIAS**<br>-Low risk of bias<br>-Moderate risk of bias<br>-High risk of bias	Low risk of bias	Low risk of bias	Low risk of bias	Cannot effectively assess the risk of bias	Cannot effectively assess the risk of bias	Low risk of bias

*** The study participants in all studies were generally stable patients with diabetes attending routine diabetes outpatient clinics, similar to what would be described in most national diabetes outpatient clinics**
